# Optimization growth performance and intestinal characteristics of broiler through the use of zeolite and bioherbal-based mycotoxin binders as feed additives

**DOI:** 10.5455/javar.2025.l882

**Published:** 2025-03-24

**Authors:** Ibrahim Ibrahim, Muhammad Halim Natsir, Osfar Sjofjan, Irfan Hadji Djunaidi, Agus Susilo, Muhaimin Rifa’i, Hafsah Hafsah

**Affiliations:** 1 Department of Animal Science, Faculty of Animal Science, Universitas Brawijaya, Malang, Indonesia; 2 Department of Biology, Faculty of Mathematics and Natural Sciences, Universitas Brawijaya, Malang, Indonesia; 3 Department of Animal Science, Faculty of Animal Science and Fisheries, Universitas Tadulako, Palu, Indonesia

**Keywords:** Mycotoxin binder, feed additives, growth performance, intestinal characteristics, broiler

## Abstract

**Objective:**

This research was designed to explore the potential of mycotoxin binders derived from zeolite and bioherbal formulations as natural feed additives to enhance growth performance and intestinal characteristics in broilers.

**Materials and Methods:**

The study utilized 320 Lohmann MB 202 broilers, sourced from PT. Japfa Comfeed Indonesia, commencing from day 1 and extending over a period of 35 days. The methodological framework employed a completely eandomized design, incorporating two factors. The primary factor analyzed was the type of feed additive, designated as Zeolite (A1) and Bioherbal (A2). The secondary factor considered was the level of mycotoxin binder inclusion in the feed, set at four increments: 0% (T1), 0.2% (T2), 0.4% (T3), and 0.6% (T4), resulting in a total of eight treatment combinations, each replicated four times. The observational metrics focused on production performance and specific intestinal characteristics of the broilers.

**Results:**

The findings indicated that while the interaction between feed type and the level of additive use did not significantly influence feed consumption, weight gain, feed conversion ratio, or villi length (*p *> 0.05), there was a notable impact on the villi surface area (*p* < 0.05) and a pronounced effect on villi count and crypt depth (*p* < 0.01).

**Conclusion:**

The study concluded that mycotoxin binders containing zeolite effectively reduce mycotoxin levels in feed, whereas bioherbal additives significantly improve intestinal health. Thus, a 0.6% inclusion level of these additives is recommended as a viable alternative to antibiotics in broiler chicken diets.

## Introduction

The poultry farming industry faces numerous challenges, including mycotoxin contamination in feed materials, which can result in significant losses to livestock production and pose potential health risks to humans. Mycotoxins in livestock products, particularly aflatoxins, can lead to various health risks in humans, including liver damage and an increased risk of liver cancer. The long-term accumulation of mycotoxins in the human body, primarily through repeated consumption of contaminated chicken products, can cause serious damage to internal organs and disturbances in the immune system [1]. Several studies estimate that mycotoxin contamination in the poultry farming industry causes economic losses exceeding 500 million rupees annually [2]. These losses are primarily due to decreased feed efficiency, reduced growth rates, and lower egg production. Globally, the economic losses due to mycotoxin contamination in agricultural commodities are estimated at 932 million USD per year [3,4].

Mycotoxin compounds like asflatoxin are made by different kinds of fungi, mostly *Aspergillus flavus* and *Aspergillus parasiticus* [5]. Mycotoxin binders are substances that are made to stick to aflatoxins in an animal’s digestive tract. This stops the animals from absorbing the toxins and damaging their organs. The use of mycotoxin binders in feed aims to reduce the risk of mycotoxin toxicity in livestock, especially in highly susceptible species such as poultry, including broiler [6].

Zeolite is a natural mineral that is known to effectively bind mycotoxin in chicken feed. This is because it has a porous crystal structure that can easily grab mycotoxin molecules, stopping chickens from absorbing them [7]. There are also chemical and electrostatic properties of zeolite that help it bind mycotoxins strongly without affecting other nutrients. In the digestive system of chickens, zeolite binds and retains mycotoxins, which are then excreted along with the feces [8]. New research shows that mycotoxin detection and binding technology has improved. For example, bioherbal compounds and modified zeolites have been made that are better at absorbing mycotoxin. These innovations enhance the efficiency of mycotoxin binding and offer more sustainable and environmentally friendly solutions [9].

The routine use of antibiotics as feed additives in poultry has heightened the risk of bacterial resistance, which can lead to diseases in humans and animals [1]. In response to these adverse effects, Indonesia implemented a ban on the use of antibiotic growth promoters (AGP) starting in January 2018, through the Regulation of the Minister of Agriculture of the Republic of Indonesia No. 14//PK.350/5/2017 [10]. This policy contrasts with the European Union, which has prohibited the use of AGP since 1997 [11]. Indonesian regulations concerning the use of mycotoxin binders and other feed additives continue to evolve in line with growing awareness of health risks. The legislation is in alignment with the European Union and the United States, which have stricter regulations on controlling mycotoxins in livestock feed, including lower maximum residue limits for mycotoxins in food products circulating in the market [12]. Herbal remedies, probiotics, prebiotics, enzymes, and phytobiotics are some of the natural feed additives that could be used instead of AGP, which is no longer allowed [13]. We anticipate that bioherbals, a blend of probiotics and phytobiotics, will optimize livestock productivity and health. Additionally, bioherbals have the potential to improve gut health through their antimicrobial and anti-inflammatory effects [14]. Commonly used herbal plants as feed additives include turmeric, ginger, moringa, papaya, and betel leaf [15].

A clear knowledge gap exists because not enough research has been done on how using zeolite as a mycotoxin binder and bioherbs as a natural feed additive can help broilers grow faster and have better gut health. Most previous studies tend to focus on one aspect, namely mycotoxin control or bioherbal effects separately, without exploring the potential combination between the two. In addition, in-depth data on optimal dosage, effects on intestinal characteristics, and long-term impacts of this combination are also still limited.

The primary goal of this research is to evaluate the effectiveness of mycotoxin binders and other natural feed additives in reducing mycotoxin contamination and enhancing poultry health and productivity. The study’s goal is to find evidence-based ways to reduce the use of harmful chemical additives like AGP by using sustainable alternatives like zeolite and bioherbal. This will encourage the poultry industry to use more natural methods. The outcomes will contribute to global efforts to improve food safety and animal welfare, addressing public health issues related to mycotoxin and antibiotic resistance. This research supports international regulatory trends and seeks to provide solutions for safer, healthier poultry production worldwide.

## Materials and Methods

### Animal Ethics Committee approval

The animal experiments in this study were approved by the Animal Care and Use Committee of Brawijaya University, with approval number 120-KEP-UB 2022 dated August 30, 2022. All procedures involving animals were carried out with high regard for animal welfare and in compliance with stringent ethical standards to ensure humane treatment. The ARRIVE guidelines were meticulously followed in the experimental design, data collection, analysis, and reporting of research findings. To keep animals from suffering too much, certain steps were taken. For example, the right anesthesia and painkillers were used during procedures, and the number of animals used was cut down by planning experiments carefully and looking at statistics.

### Research materials

This study utilized 320 Lohmann MB 202 broilers obtained from PT. Japfa Comfeed Indonesia at one day old, or Day-Old Chick (DOC), without sex differentiation (unsexed). The experimental pens used were 32 litter system pens, each measuring 100 cm × 100 cm × 60 cm, housing 10 broiler chickens equipped with feed and water containers. The heating source used during the brooder phase from day 1 to day 14 consisted of two gas heaters. Zeolite was used as a mycotoxin binder in the feed additive in this study. It was then put through a 200-mesh sieve to make zeolite particles. This size aims to address the solubility of active substances that are difficult to dissolve in the digestive tract [16]. Along with this mycotoxin binder, bioherbal feed additives like Andrographis paniculata, Piper betle, Moringa oleifera, Carica papaya, Actinomycetes, Lactic Acid Bacteria, Photosynthetic Bacteria, yeast, and fermented mold were added. The bioherbal used in this study is a product made by the Laboratory of Nutrition and Feed Science, Faculty of Animal Science, University of Brawijaya Malang, with a colony count of 1.2 × 106 CFU/ml.

The broilers received a basal diet consisting of corn, concentrate, and bran in a mesh form throughout the starter and finisher periods. This feed was formulated according to the nutritional needs of the chickens. Water and feed were provided ad libitum. The composition and nutritional content of the basal feed used in the study are presented in Table 1.

### Methods

The *in vivo* experiment in this study utilized a completely randomized design (CRD) with two factors. The first factor was the type of feed: Zeolite (A1) and Bioherbal (A2). The second factor was the level of mycotoxin binder and feed additives in the feed: 0% (T1), 0.2% (T2), 0.4% (T3), and 0.6% (T4). There were four sets of each treatment, with ten broiler chickens in each set. This gave us eight treatment combinations: A1T1 = base feed + 0% zeolite addition (control); A1T2 = base feed + 0.2% zeolite addition; A1T3 = base feed + 0.4% zeolite addition; A1T4 = base feed + 0.6% zeolite addition; A2T1 = base feed + 0% zeolite and bioherbal addition (control); A2T2 = base feed + 0.2% zeolite + bioherbal; A2T3 = base feed + 0.4% zeolite + bioherbal; and A2T4 = base feed + 0.6% zeolite + bioherbal.

We selected 1 chicken from each pen at 35 days old to observe the small intestine villi, resulting in 32 samples. In order to get samples of intestinal villi, a 3 cm section of the ileum was cut open and cleaned with physiological NaCl. The small intestine villi samples were then placed into a pot film containing a 10% formalin solution, which was labeled. We then transported the samples to the laboratory for the preparation process. To prepare the sample, the small intestine lumen was cut to a thickness of 4 μm with a microtome. The sample was then put on a slide and stained with hematoxylin and eosin. We then observed parameters such as villi number, villi length, crypt depth, and villi surface area. All examinations were conducted using a Nikon Ei microscope equipped with an Optilab Plus digital camera and Optilab Viewer image processing software at 100x magnification.

### Research variables growth performance

#### Feed intake

Feed intake is the amount of feed consumed by chickens over a specific period.

Feed Intake = ∑ Feed given (gm) – ∑ Feed remaining (gm)

#### Body weight gain (bwg)

BWG is a crucial parameter in evaluating chicken growth performance, referring to the increase in body weight over a certain period.

BWG = Final body weight (gm) − Initial body weight (gm)

#### Feed conversion ratio (FCR)

The FCR is the ratio of the amount of feed consumed by broilers to the live body weight gained over a specific period. This metric is a key indicator of the efficiency with which the birds convert feed into body mass, serving as a critical factor in assessing the economic and environmental sustainability of poultry production. A lower FCR value indicates more efficient feed conversion, implying that less feed is needed to produce a unit of chicken body weight, which is desirable in both cost management and environmental impact reduction.

FCR = Σ Feed consumed (gm) / Σ Feed consumed (gm)

**Table 1.** Composition and nutrient content of basal feed during the study.

Feed ingredients	Starter (%)	Finisher (%)
Corn* (%)	60.00	60.00
Concentrate* (%)	40.00	30.00
Rice bran* (%)	-	10,00
Nutrient content**		
Metabolizable energy (kcal)	3.161	3.203
Crude protein (%)	21.16	18.67
Crude fat (%)	5.42	4.85
Crude fiber (%)	3.31	8.42
Ash (%)	5.18	6.20
Dry matter	86.73	83.58

Notes: (*) [30]; (**) Proximate analysis by the Laboratory of Nutrition and Feed Science, Brawijaya University.

#### Income over feed cost and chick cost (IOFCC)

IOFCC is the difference between the revenue obtained from the sale of live chicken weight and the costs of feed and DOC (Day Old Chick) incurred during the research period.

IOFCC = (Body weight × Price of live chicken weight) − (Feed consumption × Feed cost + Chick Cost)

#### Intestinal characteristics

The variables measured for assessing the intestinal characteristics of broilers include:

a) Villi Height (µm): Measured as the highest distance from the base to the tip of the villus.

b) Villi Width (µm): Measured by averaging the apical width (the top width of the villus) and the basal width (the bottom width of the villus).

c) Crypt of Depth (µm): Measured as the deepest distance into the crypts beneath the villi.

d) Villi Surface Area (µm²): According to the surface area of the villi is calculated using the formula c + d/d ×a (a = the height of the villi, c = is the basal width of the villi, and d = is the apical width of the villi).

### Data analysis

Data collection in the field was conducted weekly, with the research findings recorded and organized in tables using Microsoft Excel. The data were then analyzed using Analysis of Variance based on a CRD in a factorial arrangement. If significant differences were found (*p* < 0.05) or highly significant differences (*p *< 0.01), further analysis was conducted using Duncan’s Multiple Range Test to explore the deeper differences between treatments.

## Results and Discussion

### Growth performance

To properly analyze the impact of using zeolite as a mycotoxin binder and bioherbal feed additives on growth performance (feed intake, body weight gain, feed conversion ratio, income over feed, and chick cost) and intestinal characteristics (number of villi, villi height, crypt of depth, and villi surface area), the data should be meticulously collected and organized into Tables 2 and 3, as mentioned.

### Feed intake

The statistical analysis shows that the amount of feed additives used and the type of feed do not have a big effect on the amount of feed that broiler chickens eat (*p* > 0.05). Furthermore, the use of zeolite as a mycotoxin binder significantly affects (*p *< 0.01) broiler chicken feed intake but does not show a significant difference (*p *> 0.05) when zeolite is combined with bioherbal feed additives. The data presented in Table 2 show that the lowest average feed intake was observed in treatment A2T3 (2,275.05 ± 294.68), and the highest in A1T1 (2,808.80 ± 176.99). This suggests that zeolite has a high absorption property, allowing it to bind and absorb mycotoxins in the chicken feed, and it can also help restore the chicken’s appetite, thus increasing feed intake [8]. One reason why the combination of zeolite and bioherbal might not have had a big effect is that the bioherbal feed additives might have smells or tastes that chickens don’t like, which would make them less hungry and less likely to eat. The findings may further indicate that there are other factors influencing feed consumption, such as environmental conditions, chicken health, or feed quality that were not fully controlled in this study [17].

**Table 2.** Average feed intake (g/bird), body weight gain (g/bird), feed conversion ratio (FCR), and income over feed and chick cost (IOFCC) (IDR/bird).

Treatment	Parameters			
	Feed intake (gm/bird)	Body weight gain (gm/bird)	Feed conversion ratio (gm/bird)	IOFCC (IDR/bird)
A1T1	2,808.80 ± 176.99	1,451.94 ± 32.38	0.199 ± 0.009	2,832.99 ± 999.48^d^
A1T2	2,748.40 ± 167.08	1,431.69 ± 94.38	0.192 ± 0.013	2,767.42 ± 1,380.58^b^
A1T3	2,738.08 ± 160.93	1,422.68 ± 66.01	0.198 ± 0.006	2,348.58 ± 1,256.58^a^
A1T4	2,624.68 ± 178.42	1,491.53 ± 71.79	0.181 ± 0.013	3,614.96 ± 464.44^f^
A2T1	2,579.38 ± 39.29	1,385.03 ± 98.11	0.187 ± 0.012	2,954.68 ± 1,477.39^e^
A2T2	2,616.23 ± 72.79	1,405.84 ± 60.83	0.186 ± 0.005	2,802.85 ± 754.21^c^
A2T3	2,275.05 ± 294.68	1,481.18 ± 145.25	0.163 ± 0.025	5,426.05 ± 968.08^h^
A2T4	2,430.45 ± 167.69	1,418.76 ± 48.02	0.171 ± 0.011	3,632.67 ± 1,062.62^g^

Notes: Different superscripts in the same column (a-h) indicate statistically significant differences (*p *< 0.05), and different capital letters (A-H) indicate highly significant differences (*p *< 0.01).

### Body weight gain

The research results presented in Table 2, from highest to lowest in terms of body weight gain in broilers, are as follows: A1T4 (1,491.53 ± 71.79), A2T3 (1,481.18 ± 145.25), A1T1 (1,451.94 ± 32.38), A1T2 (1,431.69 ± 94.38), A1T3 (1,422.68 ± 66.01), A2T4 (1,418.76 ± 48.02), A2T2 (1,405.84 ± 60.83), and A2T1 (1,385.03 ± 98.11). Statistical data analysis indicates that the interaction between feed type and the use of feed additive levels does not significantly affect (*p *> 0.05) the body weight gain in broilers. Similarly, the use of zeolite-based mycotoxin binders, both alone and combined with bioherbal feed additives, does not show a significant difference (*p *> 0.05) in their effect.

The insignificant effect between zeolite and bioherbal feed additives may have opposing effects that were not analyzed in this study. Zeolite can bind not only mycotoxins but also active compounds from bioherbals, thus reducing the effectiveness of bioherbals as feed additives. This may explain why the combination of the two materials did not provide significant results on body weight gain. Furthermore, this result may be associated with high cage density, which reduces the chicken’s space for movement, which causes competition for feed consumption, which can ultimately affect growth and inhibit body weight gain. Additionally, body weight gain is closely related to the chickens’ appetite [10]. Chickens with a good appetite tend to consume more feed, which ultimately supports optimal growth and body weight gain. Appetite can be influenced by factors such as feed quality, environmental conditions, chicken health, and genetic factors [19].

### Feed conversion ratio

The statistical data analysis reveals that the interaction between feed type and the level of feed additive use does not significantly affect (*p *> 0.05) the FCR in broilers. Furthermore, statistical analysis indicates that the use of zeolite as a mycotoxin binder significantly influences (*p *< 0.01) the FCR, but there is no significant difference (*p *> 0.05) from the use of zeolite combined with bioherbal feed additives. The lowest FCR was observed in the treatment A2T3 (0.163 ± 0.025) with the addition of 0.4% zeolite + bioherbal, indicating that a lower FCR value represents better efficiency, as chickens can achieve higher weight gain with less feed consumption [20]. Conversely, a higher FCR indicates lower efficiency, meaning chickens need more feed to achieve the same weight gain. Moreover, the use of feed additives such as enzymes and probiotics can influence FCR by enhancing nutrient availability, improving digestion, or boosting chicken gut health [21].

### Income over feed cost and chick cost

The statistical data analysis indicates that the interaction between feed type and the level of feed additives used significantly affects (*p *< 0.05) the IOFCC, as does the individual factor of using zeolite as a mycotoxin binder (*p *< 0.05). However, the factor of using zeolite combined with bioherbal feed additives shows no significant difference (*p *> 0.05) in its effect on IOFCC. It is observed that the IOFCC value for treatment A2T3 (5,426.05 ± 968.08) is higher, indicating better efficiency and resulting in higher revenue compared to the feed and chick costs incurred. In contrast, the lowest IOFCC value is seen in A1T3 (2,348.58 ± 1,256.58), suggesting that production costs are relatively high compared to the revenue generated. A good IOFCC reflects high income from the sale of broilers, achievable by ensuring optimal quality and quantity of production [22]. Additionally, high feed conversion efficiency, where chickens reach the desired body weight with relatively less feed, supports a favorable IOFCC. Furthermore, the selection of appropriate feed and efficient ration formulation is crucial [23].

### Intestinal characteristics

### Numbers of villi

Table 3 presents the findings on the number of villi from field experiments involving broilers observed in the laboratory. The statistical data indicate that the interaction between feed type and the use of feed additives levels significantly affects (*p *< 0.01) the number of villi. The use of zeolite as a mycotoxin binder alone does not show a significant difference (*p *> 0.05), while the combination of zeolite with bioherbal feed additives significantly affects (*p *< 0.01) the number, with the highest to lowest average values being A2T2 (84.25 ± 4.03), A1T4 (77.75 ± 13.33), A2T1 (68.50 ± 8.70), A1T2 (68.25 ± 2.06), A1T3 (63.50 ± 3.42), A1T1 (62.50 ± 7.55), A2T3 (60.00 ± 8.33), and A2T4 (54.50 ± 4.43). The number of villi in the small intestine of a broiler can be influenced by various factors, including genetics, diet, farm management, environmental conditions, and the overall health of the chickens [24]. Selecting or developing broiler chicken strains with superior genetics can play a role in determining villi characteristics, including their number. Breeding programs aim to produce chickens with better nutrient absorption capabilities [25].

### Length of villi

Table 3 also provides results on villi length, where statistical analysis shows that the interaction between feed type and the level of feed additives used does not significantly affect (*p *> 0.05) villi length. The use of zeolite as a mycotoxin binder alone does not make a significant difference (*p *> 0.05), while the combination of zeolite with bioherbal feed additives significantly influences (*p *< 0.05) the length. The highest average value is observed in A2T1 (763.98 ± 107.67) with the treatment using 0% without zeolite, indicating that longer villi in the small intestine are generally considered better as they increase the intestinal surface area, thus maximizing the absorption capabilities for nutrients from digested food [26]. With longer villi, the cells on the surface of the villi will have more area to capture and absorb nutrients, allowing the nutrient absorption process to be more efficient [18].

**Table 3.** Average number of villi, villi length (µm), Crypt of depth (µm), and Villi surface area (µm^2^).

Treatment	Parameters			
	Villi numbers (Transversal cut)	Villi lenght (µm)	Crypt of depth (µm)	Villi surface area (µm^2^)
A1T1	62.50 ± 7.55^C^	753.51 ± 87.56	212.60 ± 39.56^G^	1,466.63 ± 200.46^g^
A1T2	68.25 ± 2.06^E^	588.43 ± 65.82	149.08 ± 18.95^B^	1,328.80 ± 70.23^d^
A1T3	63.50 ± 3.42^D^	719.05 ± 107.23	278.88 ± 57.57^H^	1,552.95 ± 147.35^h^
A1T4	77.75 ± 13.33^G^	667.40 ± 105.07	190.38 ± 22.83^E^	1,413.78 ± 115.21^e^
A2T1	68.50 ± 8.70^F^	763.98 ± 107.67	209.33 ± 39.39^F^	1,433.03 ± 81.89^f^
A2T2	84.25 ± 4.03^H^	678.70 ± 45.07	164.38 ± 38.82^D^	1,229.85 ± 41.15^b^
A2T3	60.00 ± 8.33^B^	691.49 ± 65.44	147.00 ± 42.87^A^	1,131.35 ± 226.10^a^
A2T4	54.50 ± 4.43^A^	745.58 ± 45.92	149.55 ± 5.38^C^	1,317.25 ± 80.22^c^

Notes: Different superscripts in the same column (a-h) indicate statistically significant differences (*p *< 0.05), and different capital letters (A-H) indicate highly significant differences (*p *< 0.01).

### Crypt of depth

Table 3 shows the results for crypt depth from field experiments involving broilers observed in the laboratory. Statistical data indicate that the interaction between feed type and the use of feed additives levels, including the use of zeolite as a mycotoxin binder and the combination of zeolite with bioherbal feed additives, significantly affects (*p* < 0.01) crypt depth. The highest crypt depth was observed in the treatment A1T3 (278.88 ± 42.87) with the addition of 0.4% zeolite. This suggests that adequate crypt depth allows intestinal wall cells to develop and regenerate well, especially goblet cells. Goblet cells are crucial for secreting mucus as a defense barrier for villi against pathogenic bacterial attacks, which is important for ensuring the continuity of intestinal function and cell integrity [17]. Digestion and nutrient absorption processes can be enhanced when the intestinal structure, including crypts, is optimal. Enough crypt depth helps the intestines heal and can be very important for treating damage or wounds that may happen in the intestines [27].

### Surface area of villi

Table 3 presents the villi surface area from field experiments involving broilers. Statistical analysis of the interaction between feed type and the level of feed additives used shows a significant effect (*p *< 0.05). Further, the use of zeolite as a mycotoxin binder significantly influences (*p *< 0.01) the villi surface area, while the combination of zeolite with bioherbal feed additives does not show a significant difference (*p *> 0.05). The villi surface area values from highest to lowest are A1T3 (1,552.95 ± 147.35), A1T1 (1,466.63 ± 200.46), A2T1 (1,433.03 ± 81.89), A1T4 (1,413.78 ± 115.21), A1T2 (1,328.80 ± 70.23), A2T4 (1,317.25 ± 80.22), A2T2 (1,229.85 ± 41.15), and A2T3 (1,131.35 ± 226.10). This indicates that the villi surface area in the small intestine of broilers is crucial, as it plays a role in enhancing the efficiency of nutrient absorption, such as glucose, amino acids, vitamins, and minerals from digested feed [28]. A larger villi surface area allows for more efficient absorption processes, as microvilli on the intestinal villi act as absorption fields. High and wide villi, along with a large surface area, ultimately impact the growth of other body organs [29].

## Conclusion

The inclusion of zeolite in feed can mitigate the adverse effects of mycotoxins, as evidenced by the improved growth of broilers. Similarly, adding bioherbal feed additives up to 0.6% can improve the health of the small intestine, providing a viable alternative to antibiotics in broiler chicken feed. Therefore, it is recommended to incorporate these additives into feed formulations as a preventive measure against the potential contamination of broiler chicken feed by mycotoxins.

## List of abbreviations

A1, zeolit; A2, bioherbal; AGP, antibiotic growth promoters; ARRIVE, animal research: reporting of *in vivo* experiments; BWG, body weight gain; DOC, day old chick; FCR, feed conversion ratio; FI, feed intake; IOFCC, income over feed cost and chick cost; T1, treatment 1 0.0%; T2, treatment 2 0.2%; T3, treatment 3 0.4%; T4, treatment 4 0.6%.
